# Correction: Seroprevalence and associated factors of HIV and Hepatitis C in Brazilian high-security prisons: A state-wide epidemiological study

**DOI:** 10.1371/journal.pone.0260179

**Published:** 2021-11-11

**Authors:** Lirane Elize Defante Ferreto, Sthefanny Josephine Klein Ottoni Guedes, Fernando Braz Pauli, Samyra Soligo Rovani, Franciele Aní Caovilla Follador, Ana Paula Vieira, Renata Himovski Torres, Harnoldo Colares Coelho, Guilherme Welter Wendt

The second author’s name is spelled incorrectly. The correct name is: Sthefanny Josephine Klein Ottoni Guedes. The correct citation is: Defante Ferreto LE, Guedes SJKO, Braz Pauli F, Soligo Rovani S, Aní Caovilla Follador F, Paula Vieira A, et al. (2021) Seroprevalence and associated factors of HIV and Hepatitis C in Brazilian high-security prisons: A state-wide epidemiological study. PLoS ONE 16(7): e0255173. https://doi.org/10.1371/journal.pone.0255173
