# Supplementary material for: Seroprevalence and associated factors of HIV and Hepatitis C in Brazilian high-security prisons: A state-wide epidemiological study
Source: PLoS One. 2021 Jul 26;16(7):e0255173. doi: 10.1371/journal.pone.0255173 (PMC8312944; doi:10.1371/journal.pone.0255173)
Supplement: Attachment — Submitted filename: My Reviewer comments for Plos One docx.docx [file pone.0255173.s001.docx]

Reviewer comments

Manuscript ID: PONE-D-21-05227

Title" Prevalence and associated factors of HIV and Hepatitis C in Brazilian high-security

3 prisons: A state-wide epidemiological study".

Generally speaking:
Thank you for providing me the opportunity to review this manuscript that raises important critical issues about assessing the Prevalence and associated factors of HIV and Hepatitis C in Brazilian high-security 3 prisons. Such assessment will provide concrete data that will help in the micro-elimination of HCV. Definitely, the awareness about what is going on in prisoners is essential and would be great if the authors could provide in addition the situation of the action taken by the government e.g treatment options, strategy development and what is planned for this particular at risk groups

Following are my specific comments:

Comment:1

1.     Title: is specific and reflective of the research work content.

Comment:2
2.     Abstract:
a) Background –correct with a sentence focused on a key idea explaining why this topic was chosen.

b) Objectives – add the risk factors under the investigation

c) Methodology - correct

d) Results - need some quantification on the risk factors ….

e) Conclusions- are too general; the conclusion should mention specific challenges to be met as well as specific recommendations that is related to the findings of the manuscript.

Comment:3
3.     Introduction:

Well written explaining why this topic was chosen for analysis in this article.


Comment:4

Methods:

Generally, the information mentioned under the methodology deficient and should be divided into several sub-sections as follow:

a.	Type of the study

b.	Study setting

c.	Study Participants:

d.	Sample type and size: The basis of sample size calculation should be mentioned to know the confidence level and the margin of error.

e.	Measurements- OR is calculated for both the comparative cross section study and case control study as well. Please refer to the comment 5-2 in the study section.

Comment:5 Results: has to be badly improved for all the tables

5. 1    Results – should be separated from the discussion section

5.2 The mentioned sample size was 1,154 with overall prevalence of HCV and HIV detected among 1,132 meaning that only 22 cases were –ve. Accordingly, the authors has to mention how they calculated the Odds Ratio with this very small number of the comparative group. This imply that All the tables should include two columns for number and % of HCV +ve and HCV _ve and 2 columns for HIV +ve and –ve for the results to be more understandable.

5.2 line 140 and 141 indicated that the prevalence of HCV (2.7%) and HIV (1.6%) while the tables mentioned an overall prevalence 1,132: please clarify

Comment:6
6.   Discussion: should be separate section, focusing discussion on critical or essential findings and explicitly linking the conclusions with the reported data should be more emphasized.

Comment:7

7.  Conclusion – is general one, it   should be specific and explicitly linking the conclusions with the reported data, write suggestions for improvement as well as add limitations of the proposed technique to the conclusion section and provide recommendations for future research that is focused on the findings

8. References: adequate
